# Characteristics of current perception produced by intermediate-frequency contact currents in healthy adults

**DOI:** 10.3389/fnins.2023.1145505

**Published:** 2023-04-25

**Authors:** Shintaro Uehara, Akiko Yuasa, Kazuki Ushizawa, Sachiko Kodera, Yoshitsugu Kamimura, Akimasa Hirata, Yohei Otaka

**Affiliations:** ^1^Faculty of Rehabilitation, School of Health Sciences, Fujita Health University, Toyoake, Japan; ^2^Department of Rehabilitation Medicine I, School of Medicine, Fujita Health University, Toyoake, Japan; ^3^Department of Electrical and Mechanical Engineering, Nagoya Institute of Technology, Nagoya, Japan; ^4^Center of Biomedical Physics and Information Technology, Nagoya Institute of Technology, Nagoya, Japan; ^5^Department of Fundamental Engineering, School of Engineering, Utsunomiya University, Utsunomiya, Japan

**Keywords:** contact current, intermediate-frequency band, perception threshold, sensation, warmth, tingling

## Abstract

**Introduction:**

Contact electrical currents in humans stimulate peripheral nerves at frequencies of <100 kHz, producing sensations such as tingling. At frequencies above 100 kHz, heating becomes dominant, resulting in a sensation of warmth. When the current amplitude exceeds the threshold, the sensation results in discomfort or pain. In international guidelines and standards for human protection from electromagnetic fields, the limit for the contact current amplitude has been prescribed. Although the types of sensations produced by contact current at low frequencies, i.e., approximately 50–60 Hz, and the corresponding perception thresholds have been investigated, there is a lack of knowledge about those in the intermediate-frequency band—particularly from 100 kHz to 10 MHz.

**Methods:**

In this study, we investigated the current-perception threshold and types of sensations for 88 healthy adults (range: 20–79 years old) with a fingertip exposed to contact currents at 100 kHz, 300 kHz, 1 MHz, 3 MHz, and 10 MHz.

**Results:**

The current perception thresholds at frequencies ranging from 300 kHz to 10 MHz were 20–30% higher than those at 100 kHz (*p* < 0.001). In addition, a statistical analysis revealed that the perception thresholds were correlated with the age or finger circumference: older participants and those with larger finger circumferences exhibited higher thresholds. At frequencies of ≥300 kHz, the contact current mainly produced a warmth sensation, which differed from the tingling/pricking sensation produced by the current at 100 kHz.

**Discussion:**

These results indicate that there exists a transition of the produced sensations and their perception threshold between 100 kHz and 300 kHz. The findings of this study are useful for revising the international guidelines and standards for contact currents at intermediate frequencies.

**Clinical trial registration:**

https://center6.umin.ac.jp/cgi-open-bin/icdr_e/ctr_view.cgi?recptno=R000045660, identifier UMIN 000045213.

## 1. Introduction

The use of electromagnetic fields has increased considerably in the past few decades. Thus, humans are frequently exposed to electromagnetic fields emitted from various devices and systems. There are concerns regarding the adverse health effects of exposure to electromagnetic fields. According to the relevant international guidelines (International Commission on Non-Ionizing Radiation Protection, 2020) and standards (The Institute of Electrical and Electronics Engineers, 2019), the nerve activation (stimulation) is dominant at frequencies of <100 kHz, and heating is dominant at frequencies of >100 kHz. The transition frequency may be different for different exposure scenarios and sources.

The International Commission of Non-ionizing Radiation Protection (ICNIRP) revised its guidelines in 2020. One notable revision was the introduction of “guidance” for electrical current instead of the reference level in the guidelines of 1998 (International Commission on Non-Ionizing Radiation Protection, 1998). According to the revised version, “*these guidelines do not provide restrictions for contact currents, and instead provide “guidance” to assist those responsible for transmitting high-power radiofrequency fields to understand contact currents*” (International Commission on Non-Ionizing Radiation Protection, 2020).

Similar to direct exposure to electromagnetic fields, electrical currents stimulate peripheral nerves, producing various sensations, such as tingling at low frequencies and heating at high frequencies, which can result in discomfort or pain with excessive exposure (International Commission on Non-Ionizing Radiation Protection, 2020; Kavet and Tell, 2023). Above 300–400 kHz, the intensity needed for electrostimulation increases almost linearly with frequency due to the membrane response (Kavet and Tell, 2023). For simultaneous exposures at multi-frequencies or pulse exposure, their effects should be additive (International Commission on Non-Ionizing Radiation Protection, 2020), but separately for stimulation and heating. The transition frequency from stimulation to heating differs among different exposure scenarios, and few studies have been performed on this (Chatterjee et al., 1986; Nakatani-Enomoto et al., 2019). These effects are generally discussed separately from either viewpoint. In the ICNIRP Knowledge Gap document, the “pain threshold” is listed as one of the topics (Ziegelberger et al., 2020). Similarly, the research agenda of IEEE International Committee on Electromagnetic Safety includes contact current, as it is similar to external electromagnetic field exposure (Reilly and Hirata, 2016).

In addition to some attempts to understand the types of sensations induced by contact currents with a wide range of low frequency from 5 to 2,000 Hz (Félix et al., 2009; Martins et al., 2013), the types of sensations that can be induced and the stimulation intensity levels at which humans feel these sensations (i.e., perception threshold) when exposed to contact currents at low frequencies, i.e., approximately 50–60 Hz, have been widely investigated. However, to the best of our knowledge, only two studies have focused on the perception threshold at intermediate frequencies. Chatterjee et al. (1986) investigated the minimum intensity of contact currents for inducing sensations in the frequency range of 10 kHz to 3 MHz. They reported that as the frequency increased from 10 to 100 kHz, the perception threshold increased linearly, and it plateaued at higher frequencies. Furthermore, the study revealed that the produced sensations were tingling/pricking with the contact current at frequencies of <100 kHz and warmth/heat at higher frequencies (Chatterjee et al., 1986). Additionally, a recent study focused on the current perception threshold in the frequency range of 50 Hz–300 kHz and the differences between sexes and various age populations (Nakatani-Enomoto et al., 2019). The results indicated that the perception threshold has sex differences and a significant correlation with age. However, little is known about the types of sensations and their perception thresholds in the wider range of the intermediate-frequency band. Moreover, the effects of individual physical characteristics on the current perception remain unclear.

In summary, there is a significant lack of knowledge regarding (i) the transition frequency from stimulation to heating; (ii) the perception threshold in the intermediate-frequency band, i.e., 100 kHz–10 MHz; and (iii) the effects of individual physical characteristics on the perception. Thus, in the present study, we investigated the perception thresholds and types of sensations produced when contact currents in the intermediate-frequency band, i.e., at 100 kHz, 300 kHz, 1 MHz, 3 MHz, and 10 MHz, were applied to healthy adults of a wide age range.

## 2. Materials and methods

### 2.1. Participants

Eighty-eight healthy adults (44 women; mean: 49.4 years old, range: 20–79 years old; all right-handed) participated in this study. To avoid bias in ages and sex, we set age groups of 20–39, 40–59, and 60–79 years and recruited similar numbers of males and females for each group. All participants provided written informed consent before participation according to the Declaration of Helsinki of 1964, as revised in 2013. We included participants if they met the following inclusion criteria: no history of diabetes mellitus or brain, neuromuscular, or psychiatric diseases. We set the exclusion criteria as follows: (1) a history of receiving medical treatment with the potential to influence sensation or perception; (2) having a cardiac pacemaker; (3) being pregnant; (4) having eczema or skin rashes on the hand. The study protocol was approved by the Ethics Review Committee at Fujita Health University (approved no. HM20-430) and registered in the UMIN Clinical Trials Registry. All participants received payment for their participation, primarily to reimburse them for their travel and compensation for the time.

### 2.2. Equipment

**FIGURE 1:**
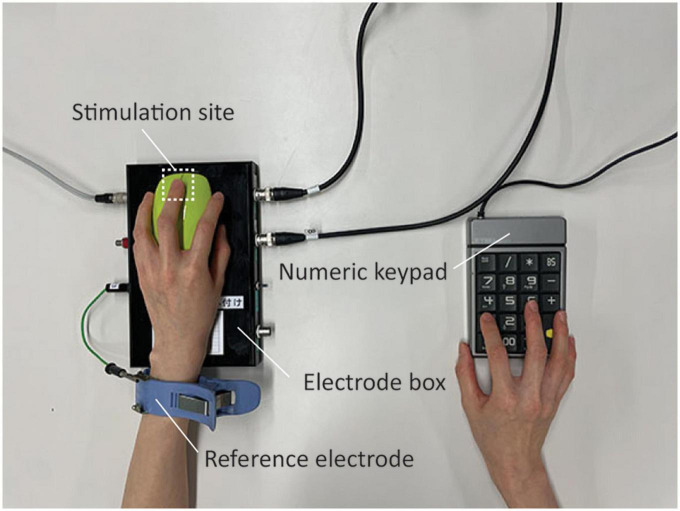
Experimental settings. Participants placed the middle fingertips of their non-dominant (left) hands on the stimulation electrode. They placed their right hands on a numeric keypad to quickly respond by pressing the 1 or 0 key to indicate the presence or absence of perceptions.

In this study, the contact current was applied to the fingertip of the left (non-dominant) middle finger, and the perception threshold and types of sensations produced were measured. We targeted the non-dominant left hand under the assumption that it would be less likely to have skin problems owing to less use in daily living. The equipment for producing the pseudo-contact current consisted of a function generator (Agilent 33210A; Agilent Technologies, Santa Clara, CA), digital multimeter (Agilent 34410A; Agilent Technologies), power amplifier (A009K251-4444R; R&K, Shizuoka, Japan), and custom-designed impedance converter and electrode box fabricated by remodeling a mouse key (Kamimura et al., 2020). The scroll wheel of the mouse key was replaced with a stimulation electrode made of brass having an area of 9 mm × 21 mm, on which the participants placed their left middle fingertips. The ground electrode was attached to the left wrist (Figure 1).

### 2.3. Experimental procedures

The experiments were performed in a quiet room with an average temperature of 23.3°C [standard deviation (SD) 2.7]. The participant sat on a chair and placed his/her left middle fingertip on the stimulation electrode. To ensure a low electrode impedance and stable current, electrode conductive cream (OJE-01D, FUKUDA COLIN, Tokyo) was applied to the fingertip and the wrist.

We started with familiarization sessions where the participants experienced typical sensations when exposed to contact currents. We selected the frequencies of 100 kHz and 3 MHz, which are known to induce tingling/pricking and warmth/heat sensations, respectively, in our preliminary investigation. In these sessions, contact currents were delivered with the intensity increasing from 0 to 100 mA stepwise in intervals of 1 s. The participant responded by pressing the “Enter” key of a numeric keypad with his/her right hand when he/she perceived a certain sensation on the fingertip. This procedure was repeated twice for 100 kHz and four times for 3 MHz: for 100 kHz, two additional sessions were performed where participants were asked to tolerate the highest possible current intensity and respond with the key press when the intensity exceeded the maximum level that they could withstand. These additional sessions were performed to familiarize the participants with stimulations producing strong sensations.

In the following experimental sessions, we used the method of constant stimuli to measure the current perception threshold (Simpson, 1988). The participants were asked whether they felt sensations when exposed to the electrical currents with different intensities. A predetermined set of 20 current intensities ranging from 2.1 to 95.0 mA was used for the stimuli. In each session, a set of stimuli was delivered in a random order. We delivered the stimulus after verbally asking the participants to prepare for the stimulus. The stimulation duration was set as 10 s at maximum, and the stimulation was terminated when the participant perceived a sensation and responded. The participants pressed “1” on the numeric keypad as soon as possible when they felt a sensation and pressed “0” if they did not feel any sensations after the 10-s stimulation. In addition, when the participants felt sensations on their fingertips, we asked them to identify the sensations as one of the following based on the previous studies (Dalziel and Mansfield, 1950; Chatterjee et al., 1986): (1) tingling/pricking, (2) warmth, (3) mixture of tingling/pricking and warmth, and (4) others. The sensation types were verbally presented to participants prior to the experimental sessions in a fixed order from (1) to (4). After the time interval of 10 s, the next stimulus with a different intensity was delivered. These procedures were performed with five stimulation frequencies (i.e., stimulation conditions): 100 kHz, 300 kHz, 1 MHz, 3 MHz, and 10 MHz. The order of the conditions was randomized among the participants. When a dummy load was connected in this system instead of a participant, we have confirmed that the temperature rise in the electrode was marginal (<0.1°C). In addition, we have confirmed measured impedance of subject with comparison of computation with a realistic finger model (Murakawa et al., 2020).

### 2.4. Determination of perception threshold

**FIGURE 2:**
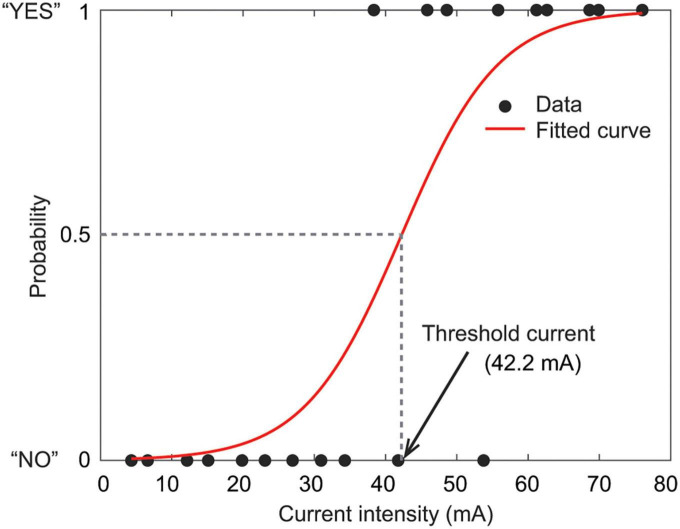
Relationship between the stimulation intensity (mA) and the presence of perception (0, no; 1, yes). Black dots represent a set of 20 stimuli with different current intensities. The red curve is the fitted psychometric function curve. The current perception threshold is defined as the stimulation intensity at which the participants were expected to feel with 50% probability.

For each stimulation condition, the relationship between the stimulation intensity (mA) and the 20 binary responses (0 or 1) was fitted with the psychometric function curve to estimate the probability of reporting sensations. The current perception threshold was defined as the stimulation intensity that the participants were expected to feel with 50% probability (Nakatani-Enomoto et al., 2019; Figure 2). If the psychometric function curve was not appropriately drawn (i.e., slope too shallow), one additional set of 20 stimuli with the same intensities was applied. If the psychometric function curve did not fit well even with these additional data, the data of this stimulation condition were excluded from the analysis.

### 2.5. Physical measurements

We measured the participants’ physical characteristics, including their body compositions, e.g., total body water and percent body fat, through bioelectrical impedance analysis using InBody270 (InBody Co., Ltd., Seoul). In addition, we manually measured the distance from the crease to the fingertip and the circumference of the distal interphalangeal joint of the left middle finger.

### 2.6. Statistical analysis

To evaluate the effects of the frequency and participants’ physical characteristics on the current perception threshold, we performed mixed-effects modeling with the frequency, age, sex, body mass index (BMI), and circumference of the distal interphalangeal joint in the left middle finger as fixed effects and with the participant as a random effect. These parameters were identified as having potential to influence the perception threshold, according to previous findings (Nakatani-Enomoto et al., 2019). To test the hypothesis that the perception threshold for current at 100 kHz would differ significantly from those at higher frequencies, we set the perception threshold at 100 kHz as the reference category.

All statistical analyses were performed with SPSS (ver. 26; IBM, Armonk, NY). Effects were considered significant if *p* was <0.05.

## 3. Results

### 3.1. Participants’ characteristics

The total number of recruited participants in the provisional age groups of 20–39, 40–59, and 60–79 years were 30, 29, and 29, respectively (Table 1). Throughout the experiment, no adverse events occurred for any participants.

**TABLE 1:** Participant characteristics.

Characteristics	Age group (years)					
	20–39 (*n* = 30)		40–59 (*n* = 29)		60–79 (*n* = 29)	
Gender						
Male, *n* (%)	15	(17.0)	13	(14.8)	16	(18.2)
Female, *n* (%)	15	(17.0)	16	(18.2)	13	(14.8)
Age, years	29.1	(20–36)	49.1	(40–59)	70.1	(60–79)
Height, cm	164.2	(149.0–180.0)	164.8	(150.0–182.0)	159.9	(148.0–173.0)
Weight, kg	58.5	(41.4–82.7)	60.8	(43.8–86.9)	60.0	(38.0–79.3)
BMI, kg/m^2^	21.6	(17.2–28.3)	22.3	(17.3–31.0)	23.3	(16.1–29.5)
Total body water, L	32.6	(22.1–46.1)	33.3	(26.3–43.8)	31.6	(22.5–39.8)
Percent body fat, %	24.0	(13.7–39.4)	24.8	(11.3–38.9)	27.7	(16.1–35.8)
Finger length^a^, cm	7.9	(6.5–9.3)	7.9	(7.5–8.8)	7.8	(7.0–8.7)
Finger circumference^b^, cm	4.8	(4.0–5.6)	5.1	(4.4–5.8)	5.4	(4.6–6.0)

Without specifying, the average and the range for the participants are presented.

^a^The distance from the crease to the tip of the left middle finger.

^b^The circumference of the distal interphalangeal joint of the left middle finger.

n, number; cm, centimeter; kg, kilogram; kg/m^2^, kilogram per square meter; L, liter; BMI, body mass index.

### 3.2. Perception threshold

Data from some participants were discarded from the main analysis because of a failure to estimate the perception threshold. Additionally, some participants’ data were not obtained for the high intensity of 100 kHz, because they could not tolerate such stimulations, owing to unpleasant sensations. In general, the number of discarded or missing data increased with the age of the participant and the current frequency (Table 2).

**TABLE 2:** Number of participants excluded.

Age group	100 kHz	300 kHz	1 MHz	3 MHz	10 MHz
20–39 (*n* = 30)	0	0	1	0	2
40–59 (*n* = 29)	1 [1]	3	3	3	3
60–79 (*n* = 29)	5 [4]	9	11	12	15

[], number of participants who could not tolerate stimulations with high intensities. Two participants practically experienced the stimulations at 100 kHz with high intensity but decided not to perform further trials with different intensities. The other three decided not to participate in the experimental sessions with the frequency of 100 kHz after experiencing stimulations in the familiarization sessions.

**FIGURE 3:**
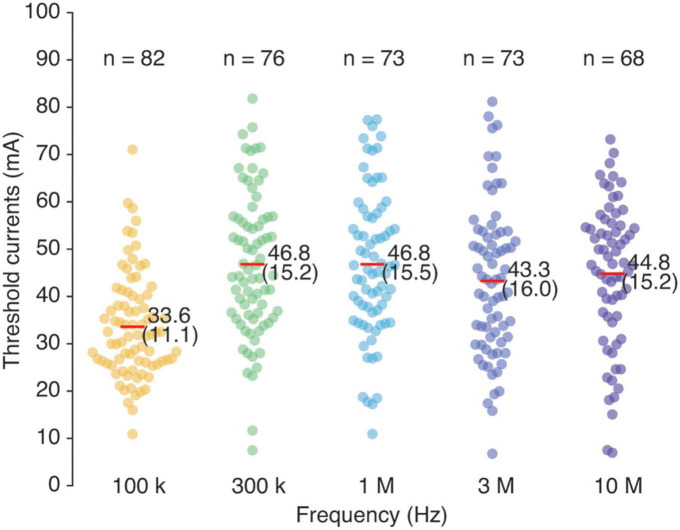
Current perception thresholds (mA) under exposure to contact currents at frequencies of 100 kHz, 300 kHz, 1 MHz, 3 MHz, and 10 MHz. Colored dots represent individual data. Red horizontal thin lines with values represent the mean (and standard deviation). The number of participants for each stimulation condition is shown at the top.

The results for the perception threshold indicated that the thresholds at frequencies of ≥300 kHz were higher than those at 100 kHz (Figure 3 and Table 3). Supporting this, the mixed-effects model revealed a significant main effect of the frequency (*F*_4,282_ = 37.4, *p* < 0.001). In particular, the thresholds at frequencies of ≥300 kHz were significantly higher than those at 100 kHz (all comparisons: *p* < 0.001). The mixed-effects model also revealed main effects of age (*F*_1,78_ = 12.1, *p* = 0.001) and finger circumference (*F*_1,77_ = 5.4, *p* = 0.023) on the perception threshold, indicating that the perception threshold was higher for older participants and participants with larger finger circumferences. No significant main effects were observed for sex (*F*_1,79_ = 1.7, *p* = 0.196) or BMI (*F*_1,76_ = 0.7, *p* = 0.423).

**TABLE 3:** Results of mixed-effects modeling for the perception threshold.

Parameters	Estimate	*p*-value	95% CI	
			Lower	Upper
Intercept	–29.34	0.050	–58.72	0.03
[*Sex*=*male*]	0^a^	–	–	–
[*Sex*=*female*]	–3.80	0.196	–9.61	2.00
[*Frequency*=100*kHz*]	0^a^	–	–	–
[*Frequency*=300*kHz*]	14.27	0.000	11.52	17.02
[*Frequency*=1*MHz*]	13.94	0.000	11.16	16.73
[*Frequency*=3*MHz*]	11.24	0.000	8.45	14.02
[*Frequency*=10*MHz*]	13.68	0.000	10.83	16.53
Age	0.27	0.001	0.11	0.42
BMI	0.33	0.423	–0.48	1.14
Finger circumference	7.97	0.023	1.15	14.78

^a^Reference category.

CI, confidence interval; BMI, body mass index.

### 3.3. Response time for perception

The response time for current perception was measured as the duration from the moment when the current was provided to the moment when the participants pressed “1” on a numeric keypad during the 10-s stimulation. The mean response times for the different frequency conditions were as follows: 0.86 s (SD 0.68) at 100 kHz, 5.81 s (SD 1.49) at 300 kHz, 5.90 s (SD 1.53) at 1 MHz, 5.89 s (SD 1.55) at 3 MHz, and 6.04 s (SD 1.71) at 10 MHz. Trials with response times of <0.4 s were excluded from the calculations because response times within such a short time window were not accurately measured, because of the data-sampling limitations of the experimental setup. These trials accounted for the mean 44.5% (SD 34.5) of trials across participants at 100 kHz but no trials at any other frequencies.

### 3.4. Types of sensations produced

**FIGURE 4:**
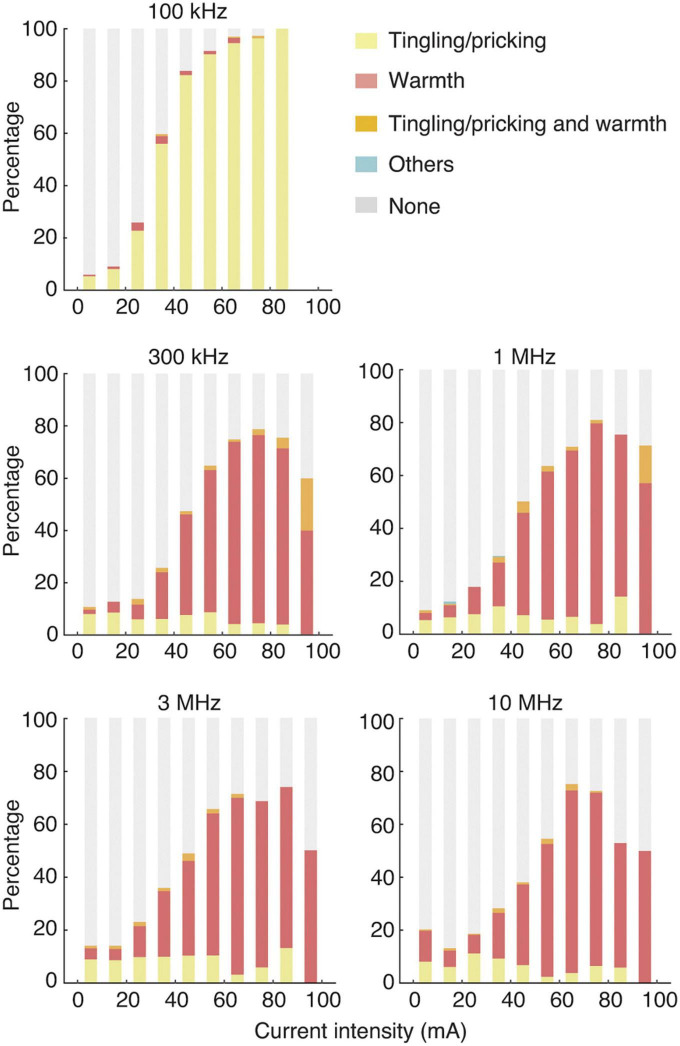
Types of sensations produced in each stimulation condition. The horizontal and vertical axes indicate the current intensity (mA) and the percentage of participants. The yellow, pink, orange, right blue, and gray bars represent tingling/pricking, warmth, a combination of tingling/prinking and warmth, others, and none, respectively. Data are displayed in bars with a bin width of 10 mA. Data were not obtained at current intensities of >90 mA under the stimulation condition of 100 kHz, because of the unpleasant sensation potentially induced by high stimulation intensities.

In accordance with the significant differences in the perception threshold currents at frequencies of ≥300 kHz compared with those at 100 kHz, the perceived sensations differed among the frequencies (Figure 4). The contact currents at frequencies of ≥300 kHz mainly produced a sensation of warmth, whereas those at 100 kHz produced a clear sensation of tingling/pricking. In all the conditions, the percentage of participants who felt a sensation tended to increase with the stimulation intensity.

## 4. Discussion

The characteristics of current perception for contact currents ranging from 100 kHz to 10 MHz were evaluated. The results indicated that the current perception thresholds and the produced sensations at ≥300 kHz were different significantly from those at 100 kHz, indicating a qualitative transition of the perception. Furthermore, the present study revealed that the current perception threshold is related to age and individual physical characteristics.

The differences in the perception threshold and the types of sensations produced between 100 and ≥300 kHz are attributed to the different types of excited sensory receptors. According to the produced sensations, the currents at 100 kHz activate free nerve ending receptors, which transmit tingling/pricking signals mainly through Aδ fibers (Hall and Hall, 2021). At frequencies higher than 300–400 Hz, the threshold current strength needed for electrostimulation increases almost linearly with an increase in the frequency, which is attributed to the shorter time available for the accumulation of electric charge on the nerve membrane (Reilly, 1998). The threshold of thermoreceptors (i.e., C warm receptor), which transmit warmth signals through C fibers (Hallin et al., 1981), would then become lower than that of electrostimulation (axon stimulation of Aδ fibers) at ≥300 kHz. The thermal sensation threshold of the thermal receptor is almost frequency-independent, suggesting the existence of a crossover frequency from stimulation to heating.

The transition of the types of sensations from 100 to 300 kHz in this study is consistent with previous findings (Dalziel and Mansfield, 1950; Chatterjee et al., 1986). The currents below 100 kHz produced a tingling/pricking sensation (Dalziel and Mansfield, 1950; Chatterjee et al., 1986), and with an increase in the frequency, the sensation changed to faint warmth (Chatterjee et al., 1986), followed by internal heating in the frequency range of 100–200 kHz and heating beyond 200 kHz (Dalziel and Mansfield, 1950). Importantly, the present study covered a wide frequency band, providing evidence that the current perception threshold and the sensations produced remain similar even for exposure to currents at higher frequencies of 10 MHz.

Interestingly, the results of the study indicated that the perception threshold increased with the age of the participant and the finger circumference. The higher perception threshold for older participants is consistent with previous studies in which the threshold was investigated using different and partly the same stimulation frequencies (Chatterjee et al., 1986; Nakatani-Enomoto et al., 2019). It is likely that age-related reductions in skin hydration, which increase the resistance of skin to electrical current, contributed to these results (Firooz et al., 2012; Messaraa et al., 2019). Other possibilities include morphological changes in the size (García-Piqueras et al., 2019) or density of epidermal receptors (Gøransson et al., 2004) and changes in the functions of peripheral nerves or in the levels of activities in the central nervous system (Wickremaratchi and Llewelyn, 2006). One potential reason for the circumference dependence is that the induced electric field was characterized in terms of the current divided by the area. In this study, the current was fixed in each trial, whereas the induced electric field may depend on the touch area as well as the touch condition. The thickness of the skin—a simpler physical feature that determines the distance from the skin surface to the receptors—may also affect the perception threshold.

In the present study, although we could not dissociate differences in the produced sensations in detail for exposure to currents of >300 kHz, the qualitative observations indicated the possibility of slight differences in the type or clarity of sensations. At 300 kHz and 1 MHz, some participants felt a combined sensation of tingling/pricking and warmth. This indicates that axons were stimulated at high intensities in addition to thermoreceptors. At ≥3 MHz, this was not observed, because of the higher threshold of axons with the increase in the frequency. The qualitative results also suggested that the clarity could be lower for sensations of warmth than those of tingling/pricking. Indeed, the response time for perception was significantly longer for exposure to high-frequency stimulations that mainly induced sensations of warmth. Furthermore, the number of discarded data increased with the current frequency (Table 2) because of failure to estimate the perception threshold, as some participants could not clearly dissociate the presence of sensations. One potential reason for this tendency is that the displacement currents at MHz frequencies cannot be neglected, in contrast to those in the kHz band, resulting in a distorted field distribution around the finger.

The threshold of “sensation” was 43.3–46.8 mA (median) and 15.1–23.8 mA (5%ile value) at ≥300 kHz. This value is more than twice the level of 20 mA recommended by the ICNIRP for the general public (International Commission on Non-Ionizing Radiation Protection, 2020). Additionally, the exposure reference level in the IEEE standard is 50.1 mA at 300 kHz (The Institute of Electrical and Electronics Engineers, 2019). In this study, the threshold of “pain” was not derived, because the skin temperature of the fingertip did not increase sufficiently to reach the point of pain perception for the injection current of 95.0 mA. For some participants, no sensation was reported even at a current amplitude of 100 mA, which is the reference level of limb current for occupational exposure in International Commission on Non-Ionizing Radiation Protection (2020).

The present study had the following limitations. First, the pressure applied by the fingertip to the stimulation electrode was not controlled. The pressure affects the size of the contact area with the electrode, which potentially induces variability of current density (Tell and Tell, 2018) and thus affects the number of receptors activated and/or the impedance of the skin. Second, it is unclear whether the perception threshold would have changed if the stimulation was applied for >10 s. In this study, the stimulation duration was limited to 10 s so that the participants would not feel fatigue from paying attention to the stimuli throughout the experiment. The perceived sensations may become clearer with prolonged exposure to the current, or they may become rather less clear as the neural response adapts to an ongoing stimulus. Third, in the present study, we did not determine the temperatures at which the participants started to feel a warmth sensation when exposed to the contact currents. Moreover, it remains unclear whether humans feel a warmth sensation because of changes in the absolute or relative temperature of the skin surface. Furthermore, the present study did not dissociate some other sensations that could be perceived through the activation of mechanoreceptors, such as squeezing, pressure, motion, and vibration, because we included these sensations in “others.” The present study targeted the non-dominant side but not the dominant side. As a previous study suggested that nerve conduction velocity is lateralized (Tan, 1993), it cannot be ruled out that there is also a lateralization of perception threshold between the dominant and non-dominant sides. These are intriguing questions that should be addressed in future studies.

## 5. Conclusion

Intermediate-frequency electromagnetic fields—particularly between 300 kHz and 10 MHz—consistently produced a warmth sensation in the exposed body part, and the current perception thresholds were significantly higher than those at 100 kHz. This finding provides insight into the effects of electromagnetic fields in the intermediate-frequency band on human perception.

## Data availability statement

The original contributions presented in this study are included in the article, further inquiries can be directed to the corresponding authors.

## Ethics statement

This study was reviewed and approved by the Ethics Review Committee at Fujita Health University. The participants provided their written informed consent to participate in this study.

## Author contributions

SU, SK, YK, AH, and YO contributed to the conception and design of the study. YK designed and assembled the experimental apparatus (stimulator). AY and KU collected the data and performed the statistical analysis. SU, AY, and KU wrote the first draft of the manuscript. SK and AH significantly revised the manuscript. All authors approved the submitted version of the manuscript.
